# Soft drink consumption among Saudi women: patterns and influencing factors

**DOI:** 10.3389/fpubh.2025.1584809

**Published:** 2025-08-25

**Authors:** Fatmah Almoayad, Abeer Salman Alzaben, Eman M. Mortada, Rasha Alshaalan, Nada Benajiba, Nahla Bawazeer, Shahd Alabdulkader

**Affiliations:** ^1^Department of Health Sciences, College of Health and Rehabilitation Sciences, Princess Nourah bint Abdulrahman University, Riyadh, Saudi Arabia; ^2^Department of Family and Community Medicine, College of Medicine, Princess Nourah bint Abdulrahman University, Riyadh, Saudi Arabia; ^3^Unité Mixte de Recherche en Nutrition et Alimentation URAC 39 (Université Ibn Tofaïl–CNESTEN), RDC-Nutrition, Kénitra, Morocco

**Keywords:** soft drinks, consumption, women health, Saudi Arabia, behavior

## Abstract

**Objective:**

Saudi Arabia has one of the highest prevalences of obesity worldwide, and excessive consumption of sweetened soft drinks significantly contributes to this. In this study, we investigated the patterns of soft drink consumption among Saudi women and identified the socio-demographic and attitudinal factors influencing these patterns.

**Design:**

We studied 1,555 Saudi women aged 20–60 years between October 2022 and March 2023. An online questionnaire was used to collect information regarding demographics, consumption patterns, attitudes towards soft drinks, and the factors influencing consumption. Chi-square tests and regression analyses were used to identify significant associations and statistical predictors of consumption frequency.

**Results:**

The respondents were categorized into infrequent soft drinks consumers (58.9%) and frequent consumers (41.1%). Significant associations of soft drink consumption patterns with age, marital status, educational level, income, and other factors were identified. Relative youth, low educational level, and low income were negatively associated with soft drink consumption, whereas a positive attitude towards soft drinks emerged as a significant predictor of higher frequency of consumption.

**Conclusion:**

The results of this study demonstrate the high prevalence of soft drink consumption by Saudi women and the complex interplay of socio-demographic factors and attitudes with consumption patterns. Public health strategies aimed at mitigating the health risks associated with excessive soft drink consumption should focus on education and awareness campaigns tailored to various demographic groups. Although predictors were identified, causal conclusions cannot be drawn due to the cross-sectional nature of the study.

## Introduction

Saudi Arabia has one of the highest prevalences of obesity in the world, with over one-third of the adult population being affected (1). Additionally, Saudi Arabia has the highest prevalence of soft drink consumption among Middle Eastern countries (2). Unhealthy dietary habits, such as the excessive consumption of sweetened soft drinks, contribute to this statistic (3–5). Soft drink consumption may adversely impact women’s health. The regular consumption of soft drinks can lead to weight gain and increase the risk of obesity, which is associated with several chronic diseases, such as type 2 diabetes and cardiovascular diseases. Soft drink consumption may also alter insulin sensitivity, which increases the risk of developing diabetes and other metabolic diseases (6–8). This is particularly concerning, given the high prevalence of undiagnosed diabetes in Saudi women 48.4%; Daoud et al. (9).

Previous studies have shown that soft drink consumption contributes to the incidences of overweight and obesity (9). Basu et al. (53) showed that a 1% increase in the consumption of soft drinks was associated with increases in weight and the prevalence of obesity in adults of 4.8 and 2.3%, respectively. The changes in eating behaviors raise the prevalence of obesity in Saudi Arabia, such as increased consumption of sweetened beverages, high-fat and heavily processed foods, and dining out, coupled with limited physical activity. The societal changes in the Middle East have contributed to the adoption of Western dietary habits and the decline of indigenous practices. In the Makkah region of Saudi Arabia, a survey revealed that out of 2,115 participants (of whom 58.5% were female), 32.8% of the population were overweight, and 23% were having obesity. Participants with obesity were more likely to consume soft drinks and fast food over three times weekly, while overweight participants reported insufficient vegetable intake. Physical activity patterns also differed, with lean individuals engaging in more walking, jogging, and strength training compared to obese individuals (10). Moreover, soft drink consumption may contribute to the development of fatty liver disease, visceral obesity, hypertension, cardiovascular disease (11), and dental caries (12). Additionally, women who consume soft drinks regularly may also be at risk of developing osteoporosis, a condition characterized by weakened bones and a higher risk of fracture. Soft drinks contain high concentrations of phosphoric acid, which can cause calcium loss from bones. Therefore, it is essential to promote healthier dietary behaviors in women and raise their awareness of the potential health risks associated with excessive soft drink consumption.

The Saudi government has implemented various regulations and policies aimed at reducing the prevalence of obesity. In 2018, a selective taxation regimen was implemented to increase the prices of soft drinks by 50% (13, 14). Between 2010 and 2017, sales of carbonated drinks significantly decreased by 57.64%, such that Saudi Arabia experienced the most substantial consumption decline of the Arab Gulf States (15). However, food selection involves complex individual choices that have various determinants, including socio-economic and cultural factors and personal attitudes (16, 17).

Soft drink advertisements and marketing campaigns often portray these beverages as stylish and enjoyable, significantly shaping consumer attitudes and behaviors (18). A cross-sectional study among students in Jeddah, Saudi Arabia, revealed that 20.3% of participants were overweight, and 13.6% were obese. Participants with obesity were more likely to purchase foods and drinks after viewing relevant social media advertisements compared to their non-obese counterparts. Additionally, frequent purchases after viewing advertisements were associated with higher consumption of potato chips, fast foods, and sugary drinks. These findings underscore the importance of regulating unhealthy food and drink advertisements on social media platforms to mitigate their influence on dietary habits, particularly among younger generations (19).

The widespread availability of soft drinks in various social settings, including universities, workplaces, family gatherings, and vending machines, further normalizes their consumption. In response to growing health concerns, the Saudi government has implemented measures to enhance public awareness and subsequently regulate consumption. For example, in Saudi Arabia, the Saudi Food and Drug Authority (SFDA) introduced mandatory caloric labelling on packed foods and drinks. Specifically, front-of-pack and back-of-pack nutrition labelling systems, such as the Multiple Traffic Light (MTL) system, were introduced to help consumers easily identify high levels of sugars, fats, and other nutrients. These efforts aim to raise awareness about the health effects of soft drinks, encouraging healthier consumption patterns.

Previous research on the knowledge, attitudes, and perceptions of soft drink consumption highlighted important insights. A cross-sectional study in Jordan demonstrated a significant positive correlation between knowledge scores and female gender, showing that women tend to be more knowledgeable about health-related information than men (20, 21). In Saudi Arabia, A secondary data analysis of the 2021 Sharik Diet and Health National Survey (SDHNS) revealed that consumption of either soft drinks or energy drinks was associated with males, younger age, lower income, and lower physical activity, as identified through multiple logistic regression analysis (14, 22). Additionally, a qualitative study in Al Madinah Province, Saudi Arabia, identified five key themes influencing soft drink consumption: taste, habit, price, environment and social context, and health concerns. Notably, increasing public awareness of the health risks associated with high-sugar drinks, driven by societal discussions on healthy eating, was found to contribute to reduced consumption. These findings underscore the importance of targeted awareness campaigns in addressing consumption behaviors.

This study is conceptually informed by constructs from the Social Cognitive Theory (SCT) and the Theory of Planned Behavior (TPB), which together offer a comprehensive lens for understanding soft drink consumption. SCT emphasizes the influence of environmental cues, social norms, observational learning, and habitual behavior on dietary choices (23). These concepts relate directly to factors identified in our study such as availability, affordability, advertising exposure, eating context (e.g., eating out or at home), watching TV, and social gatherings, all of which reinforce consumption patterns. TPB, in contrast, focuses on individual attitudes, subjective norms, and perceived behavioral control as key predictors of intention and behavior (24). In our context, attitudes such as finding soft drinks enjoyable, indispensable during meals or gatherings, and inappropriate for children reflect internalized beliefs that shape behavior. By integrating these frameworks, we interpret soft drink consumption in Saudi Arabia as a product of both individual attitudes and socially conditioned, environmentally reinforced behaviors (25, 26).

The objectives of the present study were to investigate the patterns of soft drink consumption by Saudi women and to identify the factors that influence these patterns.

## Methods

### Study design and sampling

This cross-sectional study included a sample of 1,555 Saudi women aged 20–60 years, recruited between October 2022 and March 2023 using a non-probability, convenience sampling strategy.

Participants were invited through social media platforms (Twitter, WhatsApp, Instagram) and women’s organizations, with efforts to include representation from all 13 administrative regions in Saudi Arabia.

Women with diabetes, osteoporosis, or on medically prescribed diets were excluded to reduce potential dietary confounding.

Sample size was calculated using a formula for large populations (54), yielding a minimum of 423 participants. The final sample exceeded this threshold, enhancing power and representativeness. While this method is usually applied to large populations, it was selected to ensure an adequate minimum sample size for reliable statistical analysis of the patterns of soft drink consumption among Saudi women. No multilevel or cluster sampling was employed; all data were treated at the individual level for analysis.

### Survey procedure

Data were collected via a self-administered online questionnaire. The tool was pilot-tested with 15 Saudi women to assess clarity and cultural appropriateness. Feedback informed minor refinements in question wording and layout.

Internal consistency was assessed using Cronbach’s alpha, with attitude and environment scales achieving acceptable reliability (>0.70). The questionnaire was conceptually informed by Social Cognitive Theory (SCT) and the Theory of Planned Behavior (TPB), which emphasize the influence of attitudes, perceived norms, and environmental cues on consumption behavior.

### Measures

The first section of the questionnaire was designed to collect demographic and anthropometric data, such as the participants’ age, monthly income, educational level, and family structure. Age was categorized into four groups (20–29, 30–39, 40–49, 50–59); monthly income into four groups (<5,000 SAR, 5,000–10,000 SAR, 11,000–20,000 SAR, > 20,000SAR); educational level (primary, high school, university degree, higher education); and marital status (never married, married, divorced, widowed); Region of residence in Saudi Arabia (Central, Western, Eastern, Northern, Southern). The second section focused on the participants’ soft drink consumption patterns, including the frequency and quantity of soft drinks consumed. In this study, soft drinks were defined as non-alcoholic, sweetened carbonated beverages, including carbonated sodas, energy drinks, and other non-alcoholic beverages with added sugar. This definition aligns with previous literature on soft drink taxation in Saudi Arabia, where sugar-sweetened beverages were subject to excise taxes introduced in 2017 (13–15). Participants were asked to rate their frequency of consumption on a scale from 1 to 5, where (1 = never, 2 = rarely “1–3 times a month,” 3 = sometimes “1–2 times a week,” 4 = usually “3–6 times a week,” 5 = daily). This variable was used as the primary outcome and treated as an ordinal measure in the regression model. It was not transformed into number of days.

Respondents were then asked to report the quantity they would typically consume at a time, irrespective of their reported frequency of consumption. The quantity of consumption was based on the number of cans (1 can = 330 mL) consumed at a time: (½ can, 1 can, 2 cans, or more than 2 cans). Although collected for descriptive purposes, serving size was excluded from regression analysis due to o its strong correlation with frequency and overlap in meaning. This question enabled the assessment of hypothetical consumption behavior, including responses from individuals who indicated ‘Never’ consuming soft drinks in the frequency section, to better understand potential consumption patterns. The third section was designed to assess the participants’ attitudes towards soft drinks, including the participants’ enjoyment (defined as the self-reported level of pleasure derived from consuming soft drinks), the drinks’ perceived healthiness (the participants’ belief about whether soft drinks are beneficial or harmful to health), value for money (defined as the perception of whether the cost of the soft drink matches with its quality or quantity), indispensability during meals and social gatherings (whether participants considered soft drinks essential during meals and social gatherings), and appropriateness for children (participants’ opinions on whether soft drinks are suitable for children to consume). Participants were asked to rate their agreement or disagreement with each proposition using Likert scale of 1 (strongly disagree) to 5 (strongly agree) (27). The sum of the scores for each statement was calculated, then a total attitudes toward soft drink consumption was calculated. The fourth section was designed to identify factors associated with soft drink consumption, such as consumption at social gatherings, the availability and affordability of soft drinks, soft drinks advertising, the participants’ lifestyle habits including: eating out or eating at home, soft drink consumption during watching TV. Participants were asked to rate their level of agreement or disagreement with each proposition on a Likert scale ranging from 1 (strongly disagree) to 5 (strongly agree). Before implementation, the questionnaire was pilot-tested with 15 Saudi women across two focus groups to evaluate its clarity and ease of understanding. Feedback from these sessions was utilized to make necessary adjustments before finalizing the instrument. Test–retest reliability was not explicitly assessed; however, the pilot testing and reliability analysis support the tool’s suitability for addressing the study’s objectives (28).

### Statistical analysis

Data analyzed using the Statistical Package for the Social Sciences (SPSS) version 20.0 (SPSS, Chicago, IL, USA). Descriptive analysis was performed using frequencies and percentages for qualitative data. Frequent consumers were defined as participants who reported soft drink consumption as “sometimes,” “usually,” or “daily,” while infrequent consumers were those who reported “rarely” or “never.” Spearman correlation was used between consumption patterns of soft drinks with study variables among study respondents. Hierarchical regression analysis was performed to identify statistical predictors of soft drink consumption frequency. Variables were entered in blocks: demographic characteristics in Model 1, attitudes in Model 2, and environmental/lifestyle factors in Model 3. A *p*-value < 0.05 was considered statistically significant. Although the model explores predictive associations, the cross-sectional nature of the study limits causal interpretation.

## Results

### Patterns of consumption of soft drinks by Saudi women

Total number of participants was 1,555, most of them fall within (20–29 years) of age group, married and hold a university degree. **Mo**st of the participants (40.9%) reported that they rarely consumed soft drinks, defined as 1–3 times per month, 18% reported that they did not consume soft drinks at all, and only 5% reported daily consumption. With respect to the quantity of soft drinks consumed at a time, approximately 39% of the participants consumed 1 can, another 39% consumed 2 cans, 16% consumed less than 1 can, and 6% more than 3 cans (Table 1).

**Table 1:** Socio-demographic characteristics of the participants and patterns of soft drink consumption (*n* = 1,555).

Socio-demographic characteristics		Number	%
Age (years)	20–29	591	38.0
	30–39	532	34.2
	40–49	273	17.6
	50–59	159	10.2
Marital status	Never married	567	36.5
	Married	884	56.8
	Divorced	78	5.0
	Widowed	26	1.7
Educational level	Primary school	8	0.5
	High school	198	12.7
	University degree	1,108	71.3
	Higher level studies (master’s or PhD)	241	15.5
Monthly income (SAR)	<5,000 SAR	694	44.6
	5,000–10,000 SAR	365	23.5
	11,000–20,000 SAR	331	21.3
	> 20,000 SAR	165	10.6
Soft drink consumption frequency			
Infrequent consumption	Never	280	18.0
	Rarely (1–3 times/month)	637	40.9
		917	58.9
Frequent consumption	Sometimes (1–2 times/week)	373	23.9
	Usually (3–6 times/week)	187	12.02
	Daily	78	5.00
		638	41.1
Quantity of soft drink consumption at a time	<1 can	249	16.0
	1 can	605	39.0
	2 cans	605	39.0
	>3 cans	93	6.0
	Total	1555	100.0

SAR, Saudi Arabian riyal.

### Factors influencing soft drink consumption

The analysis of the relationship between participants’ socio-demographic characteristics and their frequency of soft drink consumption of soft drinks revealed a statistically significant associations with age, marital status, educational level, and monthly income (χ^2^ = 29.4, *p* < 0.001; χ^2^ = 19.2, *p* < 0.001; χ^2^ = 9.8, *p* = 0.02 and χ^2^ = 8.9, *p* = 0.03; respectively) (Table 2).

**Table 2:** Relationships of the frequency of soft drinks consumption with the participants’ socio-demographic characteristics (*n* = 1,555).

Characteristic	Category	frequency of soft drinks consumption		Overall no (%)		*p*-value
		Infrequent consumer no. (%)	Frequent consumer no. (%)			
Age group (years)	≤ 30	308 (33.6)	283 (44.4)	591 (38.0)	29.4	**<0.001**
	30–39	317 (34.6)	215 (33.7)	532 (34.2)		
	40–49	174 (19.0)	99 (15.5)	273 (17.6)		
	≥ 50	118 (12.9)	41 (6.4)	159 (10.2)		
Marital status	Never married	297 (32.4)	270 (42.3)	567 (36.5)	19.2	**<0.001**
	Married	560 (61.1)	324 (50.8)	884 (56.8)		
	Divorced	42 (4.6)	36 (5.6)	78 (5.0)		
	Widowed	18 (2.0)	8 (1.3)	26 (1.7)		
Educational level	Primary school	4 (0.4)	4 (0.6)	8 (0.5)	9.8	**0.02**
	High school	108 (11.8)	90 (14.1)	198 (12.7)		
	University degree	642 (70.0)	466 (73.0)	1,108 (71.3)		
	Higher studies	163 (17.8)	78 (12.2)	241 (15.5)		
Monthly income (SAR)	<5,000	389 (42.4)	305 (47.8)	694 (44.6)	8.9	**0.03**
	5,000–10,000	213 (23.2)	152 (23.8)	365 (23.5)		
	11,000–20,000	218 (23.8)	113 (17.7)	331 (21.3)		
	> 20,000	97 (10.6)	68 (10.7)	165 (10.6)		
Region of residence in Saudi Arabia	Central	410 (50.3)	274 (49.5)	684 (50.0)	9.04	0.06
	Western	184 (22.6)	122 (22.0)	306 (22.4)		
	Eastern	122 (15.0)	74 (13.4)	196 (14.3)		
	Northern	32 (3.9)	14 (2.5)	46 (3.4)		
	Southern	67 (8.2)	70 (12.6)	137 (10.0)		
Total		917 (58.9)	638 (41.1)	1,555 (100.0)		

*p-value is statistically significant < 0.05.

The factors associated with soft drink consumption was compared between frequent and infrequent consumers. Compared to infrequent consumers, frequent consumers exhibited higher scores for consumption at social gatherings for consumption at social gatherings (4 vs. 3, *p* < 0.001), the availability and affordability of soft drinks (4 vs. 3, *p* < 0.001) respectively, soft drinks advertising (4 vs. 3, *p* < 0.001), the participants’ lifestyle habits (3 vs. 2, *p* < 0.001), eating out (4 vs. 3, *p* < 0.001), and eating at home (4 vs. 4, *p* < 0.001) (Table 3a).

**Table 3:** Comparison of the factors associated with soft drink consumption and attitudes between frequent and infrequent consumers (*n* = 1,555).

Factors and attitudes related to soft drink consumption	Study group				*p*-value
	Infrequent consumers N = 917		Frequent consumers *N* = 638		
	Mdn (IQR)	Mean rank	Mdn (IQR)	Mean rank	
(a) Factors associated with soft drink consumption					
Social gatherings	3 (2)	674.56	4 (1)	926.68	<0.001*
Availability	3 (2)	675.25	4 (1)	925.68	<0.001*
Affordability	3 (2)	675.90	4 (1)	924.75	<0.001*
Advertising	3 (2)	696.51	4 (2)	895.12	<0.001*
Habits	2 (3)	710.68	3 (2)	874.76	<0.001*
Eating out	2 (3)	683.99	3 (2)	913.12	<0.001*
Eating at home	4 (1)	673.96	4 (1)	927.54	<0.001*
Watching TV	2 (3)	856.79	3 (2)	1106.14	<0.001*
(b) Attitudes towards soft drink consumption					
Very healthy	1 (0)	704.94	1 (1)	883.01	<0.001*
Enjoyable	2 (3)	605.48	4 (2)	1,025.97	<0.001*
Value for money	2 (2)	746.71	2 (1)	822.97	0.01*
Indispensable during meals	1 (1)	582.39	2 (2)	1,059.15	<0.001*
Indispensable during social gatherings	4 (1)	810.18	4 (2)	731.74	<0.001*
Inappropriate for children	2 (3)	641.24	4 (2)	974.57	<0.001*
Overall attitude score Mean (SD)	14.1 (3.09)		17.9 (3.8)		<0.001*

U, Mann–Whitney test; T, Student’s t-test; SD, standard deviation; Mdn, median; IQR, interquartile range. *p-value is statistically significant < 0.05.

Moreover, the differences between frequent and infrequent consumers regarding their attitudes towards soft drinks were significant. Compared to infrequent consumers, frequent consumers exhibited higher scores in the participants’ enjoyment and the drinks’ perceived healthiness, value for money, indispensability during meals and social gatherings, and appropriateness for children (Table 3b).

Spearman’s correlation was utilized to analyse the relationships between patterns of consumption, participant’s characteristics and factors associated with soft drink consumption. The patterns of soft drink consumption significantly positively correlated with marital status, gatherings with families and friends, availability, affordability, advertising, habits, eating out, eating at home, and the overall attitude scores (r = 0.29, 95% CI [0.24, 0.33], r = 0.3, 95% CI [0.25, 0.34], r = 0.29, 95% CI [0.24, 0.33], r = 0.18, 95% CI [0.13, 0.23], r = 0.24, 95% CI [0.19, 0.29], r = 0.28, 95% CI [0.23, 0.33], and r = 0.53, 95% CI [0.49, 0.56], respectively; *p* < 0.001). In addition, patterns of soft drink consumption significantly negatively correlated with age, educational level, and income (r = −0.14, 95% CI [−0.19, −0.091], r = 0.07, 95% CI [0.02, 0.12]. and r = 0.05, 95% CI [0.00029, 0.099], respectively, *p* < 0.001, 0.002 and 0.04) (Table 4).

**Table 4:** Correlation matrix of patterns of soft drink consumption, participants characteristics and factors associated with soft drink consumption among Saudi women.

Variable	Correlation coefficient	95% CI	*p*-value
Age	−0.14**	−0.19, −0.091	<0.001
Marital status	−0.08**	0.03, 0.13	0.002
Educational level	−0.07**	0.02, 0.12	0.002
Income	−0.05*	0.00029, 0.099	0.04
Region of residence	0.04	0.13, 0.23	0.18
Social gatherings	0.29**	0.24, 0.33	<0.001
Availability	0.3**	0.25, 0.34	<0.001
Affordability	0.29**	0.24, 0.33	<0.001
Advertising	0.18**	0.13, 0.23	<0.001
Habits	0.24**	0.19, 0.29	<0.001
Eating out	0.28**	0.23, 0.33	<0.001
Eating at home	0.28**	0.23, 0.33	<0.001
Overall attitude	0.53**	0.49, 0.56	<0.001

*Correlation is significant at *p* < 0.05; **Correlation is significant at *p* < 0.01.

Table 5 shows the results of the hierarchical regression analysis use to identify the predictors of soft drink consumption frequency. In this table, the first model included the sociodemographic characteristics (age, marital status, educational level, income), the second model included the sociodemographic characteristics, and the factors associated with soft drink consumption (social gatherings, availability, affordability, advertising, habits, eating out, and eating at home), and the third model included the sociodemographic characteristics, and the factors associated with soft drink consumption and the overall attitude. The multiple regression model revealed that the third model was the best predictor of a participant’s soft drink consumption and predicted 33% of the variance in the frequency of soft drink consumption (*R*^2^ = 0.33, *p <* 0.01; Table 5). Of the 12 predicting variables, four were found to significantly predict soft drink consumption: educational level (ß = −0.087, *t* = −3.86, *p <* 0.001), eating out (ß = 0.06, *t* = 2.31, *p =* 0.021), eating at home (ß = 0.16, *t* = 7.47, *p <* 0.001), and overall attitude (ß = −0.4, *t* = 17.30, *p* < 0.001). These findings suggest that demographic, behavioral, and attitudinal factors are statistically associated with the frequency of soft drink consumption among Saudi women. While these variables statistically predict consumption within the model, the cross-sectional nature of the study does not permit causal inference.

**Table 5:** Regression analysis results for identifying predictors of soft drink consumption.

Predictor variables^d^	Model 1^a^				Model 2^b^				Model 3^c^			
	B	95% Confidence interval for B		*P*	B	95% Confidence interval for B		*P*	B	95% Confidence interval for B		*p*
		Lower bound	Upper bound			Lower bound	Upper bound			Lower bound	Upper bound	
Constant	0.76	0.61	0.91	<0.001*	−0.09	−0.15	0.14	0.254	−0.64	−0.79	−0.47	<0.001*
Age	−0.07	−0.10	−0.04	<0.001*	−0.05	−0.26	0.07	0.002*	−0.02	−0.05	0.01	0.18
Marital status	−0.01	−0.05	0.04	0.8	0.01	−0.08	−0.02	0.794	0.01	−0.03	0.05	0.49
Education	−0.08	−0.12	−0.03	0.002*	−0.08	−0.04	0.05	0.001*	−0.08	−0.12	−0.04	<0.001*
Income	0.02	−0.01	0.04	0.26	0.004	−0.12	−0.03	0.76	−0.01	−0.03	0.02	0.56
Gatherings					0.041	−0.02	0.03	0.001*	−0.01	−0.03	0.01	0.358
Availability					0.025	0.02	0.07	0.07	0.02	−0.007	0.04	0.169
Affordability					0.042	−0.002	0.05	0.001*	0.02	−0.002	0.04	0.079
Advertising					−0.012	0.02	0.07	0.265	−0.001	−0.02	0.02	0.903
Habits					0.024	−0.03	0.01	0.03*	0.01	−0.01	0.03	0.550
Eating out					0.045	0.002	0.05	<0.001*	0.03	0.005	0.05	0.021
Eating in					0.088	0.02	0.07	<0.001*	0.06	0.05	0.09	<0.001*
Attitude									0.06	−0.03	0.01	<0.001*
Model statistics												
*F*	6.864*				30.34*				51.3*			
*R* ^2^	0.025				0.21				0.33			
△*R*^2^	0.025				0.19				0.11			

*p-value is statistically significant < 0.05. B, non-standardized beta regression coefficient; F, ANOVA value; △*R*^2^, change in *R*^2^; *R*^2^, coefficient of determination.

a Predictors: (Constant), age, marital status, educational level, income, region of residence.

b Predictors: (Constant), age, marital status, educational level, income, region of residence, social gatherings, availability, affordability, advertising, habits, eating out, eating at home.

c Predictors: (Constant), age, marital status, educational level, income, region of residence, social gatherings, availability, affordability, advertising, habits, eating out, eating at home, overall attitude.

^d^Dependent variable: patterns of soft drink.

## Discussion

The aim of the present study was to investigate and identify the factors influencing soft drink consumption in Saudi Arabian women. Eighty-two percent of the sample consumed soft drinks, with about 41% reporting that they rarely consumed them (1–3 times per month). The high prevalence of soft drink consumption among these women is consistent with published evidence that Saudi Arabia is the largest consumer of soft drinks in the Middle East region. Specifically, Saudi Arabia is ranked fifth in terms of the number of calories derived from sugar-sweetened beverages (2). The present findings are consistent with those of previous studies of Saudi adults, which identified prevalences of soft drink consumption of 67 and 86% (6, 22).

In terms of the frequency and quantity of soft drinks consumed, out of the total sample, only 5% of the participants consumed soft drinks daily, approximately 39% of the total sample consumed one can at a time, and another 39% consumed two cans at a time. While these frequencies are lower than those reported for other countries, such as the UK (20.4%) (29), the USA (40.0%) (30), and South Africa (48.3%) (31), a significant proportion consumed them weekly, with 23.9% consuming 1–2 times per week and 12% consuming 3–6 times per week. Similarly, secondary data from the 2021 Sharik Diet and Health National Survey (SDHNS) indicated that 67% of participants consumed soft drinks weekly. The low daily soft drink consumption in Saudi Arabia may be attributable to the implementation of a 50% tax on soft drinks, which significantly raised their prices (13–15). Previous studies have emphasized the importance of the frequency of consumption rather than the quantity; however, reducing the frequency of consumption can significantly reduce the overall quantity consumed as well as the associated health risks (28, 32).

In the present study, we identified several factors that influence the soft drink consumption patterns among women in Saudi Arabia. We identified significant positive correlations of soft drink consumption with marital status, gatherings with family and friends, availability, affordability, advertising, habits, eating out, eating at home, and overall attitude. Our findings of positive associations of soft drink consumption with social gatherings, eating out, and eating at home are consistent with those of previous studies (28, 33).

In the Middle eastern countries generally and Saudi culture specifically, sharing beverages—including soft drinks—during family and social gatherings forms part of broader hospitality traditions, reinforcing their availability and social acceptability in both home and public settings (34, 35). Hence, eating at home is associated with more access to unlimited servings and larger portion sizes, which can contribute to higher consumption. Furthermore, a study of female students reported that such beverages were commonly consumed during social occasions (36).

The observed relationships between soft drink consumption and social events, as well as dining behaviors, confirms the cultural integration of soft drinks in Saudi cultural practices. Future interventions should address these deeply ingrained practices by promoting culturally acceptable and healthier alternative beverages, such as infused water, herbal teas, or low-sugar drinks, alongside education on portion control and substitution behavior to reduce overall consumption. Integrating Social Norms Theory with Social Cognitive Theory (SCT) such as observational learning, outcome expectations, and reinforcement may enhance alignment with culturally rooted community expectations and support more sustainable shifts in dietary behavior (37, 38).

High availability and affordability of soft drinks were also found to be positively associated with high consumption rates. These findings suggest that the availability and affordability of soft drinks influence consumption behavior. For example, having soft drinks readily available at home or in nearby stores can increase consumption, as shown in studies by Bere et al. (39) and van der Horst et al. (40). Specifically, van der Horst et al. (40) reported an inverse relationship between soft drink consumption and proximity to the nearest shop, with consumption increasing if there was a shop within 200–300 m. Our finding that affordability plays a significant role in soft drink consumption patterns is also similar to those of other studies (6, 41).

Advertising was also found to influence consumption. We found that exposure to advertisements was positively associated with soft drink consumption, which is consistent with the findings of a previous study of Saudi adults (6). In the Saudi context, soft drink marketing often appeals to emotional and social dimensions of consumption, portraying these beverages as symbols of modernity, enjoyment, and social connection. This strategy aligns with global evidence on multi-sensory marketing’s influence on consumer behavior (18), and is reinforced by local studies highlighting the impact of advertising on young Saudi consumers’ purchasing decisions and consumption patterns (6, 19).

When we compared frequent and infrequent consumers of soft drinks, we found that frequent consumers were significantly more influenced by factors such as social gatherings, availability, affordability, and media usage. Therefore, a deeper understanding of these factors could assist in the development of targeted strategies for use in educational campaigns to effectively address consumption behaviors.

We also identified significant negative correlations between soft drink consumption and the participants’ age, educational level, and income. Younger adults (aged 30–39 years) were more likely to consume soft drinks than older adults. This is consistent with the findings of previous studies, which showed that consumption peaks in 18–39-year-olds and then declines with age (18, 30, 42, 43). However, a study of Cambodian adults showed that the highest consumption was by individuals aged 31–59 years, which may be attributable to the higher incomes of older adults (44).

Several previous studies have shown that low educational attainment is associated with higher soft drink consumption (30, 43, 45). However, one study of Saudi adults did not identify a relationship between educational level and soft drink consumption (22). We found that monthly income was significantly negatively correlated with soft drink consumption, which is consistent with previous findings reported in Saudi Arabia (42). This could be because high income and educational level may be associated with increased access to knowledge and resources that encourage healthy lifestyle choices.

It is also important to note potential differences in soft drink consumption patterns between women and men. While the present study focuses exclusively on women, previous research in Saudi Arabia and the wider Middle East region has shown that men typically report higher overall consumption of sugar-sweetened beverages, often driven by differences in lifestyle, occupational activity, and health awareness (22, 46). However, women may be more affected by social and cultural pressures related to body image, which could influence their dietary choices and openness to health-promotion messages (47). Acknowledging these gender-based differences is essential for designing effective, tailored public health interventions.

In addition to the focus on reducing soft drink consumption, public health strategies in Saudi Arabia also promote active lifestyles through community-based programs, accessible sports facilities, and awareness campaigns (48). Health-promoting activities such as regular exercise, walking, and yoga play an important role in the prevention and management of obesity and can moderate cravings for sugar-rich foods and beverages (49–51). By combining dietary interventions with the promotion of physical activity, these multifaceted strategies could more effectively address the rising prevalence of obesity and associated non-communicable diseases (52).

The present study had several strengths, including its large sample size and representation of various regions of Saudi Arabia. However, its limitations included the reliance on self-reported data collected through an online survey, which may have influenced the accuracy of the results. The use of an online survey facilitated cost-effectiveness and access to a broad geographic area. However, this method limited the generalizability of the results, particularly in reaching less-educated and rural populations who may have limited internet access or digital literacy. Future studies may consider using data collection strategies to include broader populations and compare participants’ demographics to national statistics. Additionally, future studies may consider examining the consumption patterns of groups with special health conditions or on special diets separately to better understand how health conditions influence soft drink consumption behavior. Additionally, one limitation is that the consumption quantity question was answered by all participants, including those who reported never consuming soft drinks. While intended to capture hypothetical behavior, this may have led to inconsistencies and should be considered when interpreting the quantity data. A final key limitation of this study is its cross-sectional design, which restricts the ability to infer causality or temporal relationships between variables. While regression analysis was used to explore associations, we have clearly noted throughout the manuscript that findings are associative and that the use of predictive terminology does not imply causation. Longitudinal studies are needed to confirm causality. The present findings highlight the importance of designing targeted strategies and awareness campaigns regarding soft drink consumption. Despite the government’s efforts to reduce soft drink consumption through taxation, it is critical to raise consumers’ awareness of the associated health risks. Such strategies should include awareness campaigns and intervention programs tailored to women and based on a thorough understanding of the factors influencing soft drink consumption in this population.

## Conclusion

In the present study, we performed a comprehensive analysis of the patterns of soft drinks consumption among Saudi women and its association with the socio-demographic and factors. The high prevalence of soft drink consumption by the participants highlights the need for targeted public health interventions. Young women and those with lower educational attainment and income levels are more likely to consume soft drinks frequently, suggesting that tailored education and awareness campaigns are crucial to addressing this issue. The positive associations of soft drink consumption with factors such as social gatherings, availability, affordability, and advertising indicate the need for multifaceted strategies that consider these factors. By promoting healthier dietary behaviours and raising awareness of the potential health risks associated with soft drink consumption, public health initiatives should be able to reduce the consumption of soft drinks and improve the overall health of Saudi women.

## Data Availability

The raw data supporting the conclusions of this article will be made available by the authors, without undue reservation.
